# Socioeconomic deprivation and the clinical management of self-harm: a small area analysis

**DOI:** 10.1007/s00127-017-1438-1

**Published:** 2017-10-04

**Authors:** Robert Carroll, Duleeka Knipe, Paul Moran, David Gunnell

**Affiliations:** 1 School of Social and Community Medicine, University of Bristol, Canynge Hall, 39 Whatley Road, Bristol, BS8 2PS UK; 2 Evidera, London, UK; 3 Liaison Psychiatry, Bristol Royal Infirmary, Bristol, UK

**Keywords:** Self-harm, Deprivation, Clinical care, Socioeconomic deprivation, Epidemiology

## Abstract

**Purpose:**

Socioeconomic deprivation is associated with increased rates of self-harm but its association with levels of clinical care has not previously been explored. The aim of the current study was to investigate socioeconomic differences in the clinical management of people who self-harm.

**Methods:**

Cross-sectional analysis of 3607 people presenting to a large inner-city hospital following self-harm.

**Results:**

People living in the least deprived quintile were more likely to receive a psychosocial assessment (most vs. least deprived: 63.51 vs. 70.14%). This effect persisted in our fully adjusted model (OR 1.45, CI 1.15–1.82, *p* = 0.002). Mediation analysis suggested this association was in large part explained by higher rates of self-discharge in people presenting from areas of higher deprivation.

**Conclusions:**

Compared to those from more deprived areas, people from less deprived areas are more likely to receive a psychosocial assessment when presenting to hospital following self-harm. The occurrence of higher rates of self-discharge from emergency departments among those from more deprived areas may explain the association.

**Electronic supplementary material:**

The online version of this article (doi:10.1007/s00127-017-1438-1) contains supplementary material, which is available to authorized users.

## Introduction

One in six people who die by suicide present to hospital following an episode of self-harm in the year leading up to their death [1]. Ensuring the appropriate clinical care for these people is, therefore, a key priority for suicide prevention. NICE guidelines recommend that all people presenting to hospital following self-harm should receive a psychosocial assessment and there is a growing body of evidence suggesting that such assessment, as well as facilitating referral to community services, may reduce the risk of repeat self-harm [2–4].

Socioeconomic deprivation has a well-established association with variation in clinical practice in a number of disease areas [5–7] and is strongly associated with the incidence of self-harm. However, we are not aware of any previous study investigating the relationship between level of socioeconomic deprivation and the provision of clinical care to self-harm patients. We used prospectively collected registry data to investigate whether the provision of key elements of clinical care for self-harm varies according to area-level deprivation status.

## Methods

### Bristol Self-Harm Surveillance Register

The current study was based on a consecutive series of people who presented to an inner-city hospital in South West England following self-harm between September 2010 and December 2015. A patient’s first episode of self-harm during this time period was used in the analysis. For the purposes of data collection, self-harm was defined as ‘intentional self-poisoning or self-injury, irrespective of the apparent purpose of the act’ [8]. The details of these self-harm presentations were recorded on the Bristol Self-Harm Surveillance Register [9]. Information on the register is recorded prospectively and includes details regarding patient characteristics and the clinical care they received. Potential cases recorded on the register are identified through searches of emergency department electronic records for key terms such as “overdose” or “laceration”. The medical notes of potential cases are then reviewed. If a self-harm presentation is identified, information from the medical notes, liaison psychiatry service records, the hospital patient administration system (PAS) data and the local community mental health services PAS data are retrieved. Recent audit involving a manual review of the medical notes of all ED presentation indicates that the register captures 98% of self-harm presentations.

### Clinical care

We investigated the following aspects of care (1) admission to medical bed, (2) psychosocial assessment, (3) referral to community mental health services and (4) psychiatric inpatient admission. Psychosocial assessment refers to an evaluation of needs and risks of the patient, is undertaken by specialist mental health professional, most commonly by a member of the hospital liaison psychiatry service, and is recommended for all self-harm patients by NICE [8]. The liaison psychiatry service that performed the psychosocial assessments was composed of psychiatric liaison nurses, consultant psychiatrists and junior doctors. The team’s availability varied over the study period (the service increased from a 5- to 7-day service in 2014 and increased from providing cover between from 9 a.m. to 5 p.m. to availability between 0800 and 2200; out-of-hours assessments are provided by on-call psychiatrists and community mental health teams. Patients were referred for psychosocial assessment following an initial assessment by medical colleagues at triage.

Information on patient management was retrieved from emergency department attendance cards and medical notes. Evidence of psychosocial assessment was obtained from a review of the medical notes and the records of the liaison psychiatry service, which included information on all of the patients the liaison psychiatry service assessed. Receipt of these elements of care was recorded as binary (yes/no) variables.

Information on the postcode of residence of people presenting to the hospital for self-harm is recorded on the register. These data were linked to the lower super output area (LSOA). LSOA are small areas (or neighbourhoods) which have populations ranging from 1000 to 3000 individuals. The analysis focused on the people from communities served by the hospital (population of roughly 430,000) and excluded presentations (*n* = 86) made by people living in LSOA outside this area. Sensitivity analysis were undertaken to assess the impact of this exclusion. This analysis focused on people presenting for self-harm to one of two emergency departments within the city of Bristol. Patients may have presented for self-harm at other hospitals and these presentations would not be captured by the register.

Levels of deprivation were assessed using the English index of multiple deprivation (IMD) 2015 score for each LSOA. The IMD is a relative measure of socioeconomic deprivation based on seven separate domains: income; employment; education, skills and training; health and disability; crime; barriers to housing and services; and living environment. The IMD score was categorised into quintiles with 1 being the most deprived and 5 being the least deprived.

In addition to area level of deprivation, individual characteristics were also investigated including the patient’s gender, age (10 year age bands), history of previous self-harm and method of self-harm. Methods of self-harm were categorised as either self-poisoning, self-injury or both self-poisoning and self-injury. Self-injury includes mostly self-cutting, but also rarer high lethality methods of self-harm including hanging and jumping from a height.

### Statistical analysis

The first (index) presentation on a person made for self-harm in the study period was used in the analysis. Basic descriptive statistics including Chi-squared and *t* tests were used to evaluate differences in the characteristics of patients across quintiles of deprivation. The main outcome of interest was whether a patient received one of the four main elements of care previously described. The odds of receipt of these elements of care were evaluated across quintiles of deprivation using logistic regression. The exposure of interest, quintile of deprivation, was treated as a categorical variable and also entered as a continuous variable in the model to test for the linear association between IMD quintile and rates of receipt of clinical care. Our analysis combined area-level deprivation with individual level patient characteristics. We therefore implemented robust standard errors to account for clustering at the LSOA level. Robust standard errors produce more conservative confidence intervals as patients who live near one another may be more likely to share similar characteristics.

Three sensitivity analyses were undertaken to evaluate the validity of our main analysis. First, we assessed the mediating effect of self-discharge on our estimates using the Stata command “binary_mediation”. Both the direct and indirect estimates were estimated along with bootstrapped standard errors and confidence intervals (500 repetitions). Second, we used multiple imputations with 20 repetitions using chained equations to assess the impact of the exclusion of patients with missing data (13.9%, 581/4188) from our main analysis. This was implemented via the user written “ice” command in Stata. Finally, we re-ran our analysis and included those patients presenting from outside the communities served by the hospital to see if this altered our findings. All analyses were undertaken using Stata (Release 14. College Station, TX: StataCorp LP.).

### Ethical approval

Ethical approval for the register was obtained from the NHS Health Research Authority via NRES Committee South West—Central Bristol.

## Results

### Cohort characteristics

Between September 2010 and December 2015, 4581 people presented to hospital following self-harm. Of these patients, 4188 (91.4%) had postcode information that could be linked to a LSOA within the study catchment. Of these, 3674 remained once those with missing data on age (10, 0.2%), gender (18, 0.4%) previous self-harm (463, 11.1%) and method of self-harm (23, 0.5%) were excluded. A further 67 (1.6%) people were excluded due to missing outcome data (psychosocial assessment, medical admission, referral to community mental health teams and psychiatric inpatient admission). Following these exclusions, a cohort of 3607 patients remained and formed the primary analytic sample.

The median age of the cohort was 30 (range 16–96). Females presented more commonly than males (59.4%, 2141/3607). Self-poisoning was the most frequent method of self-harm (76.4%, 2758/3607) followed by self-injury (16.9%, 609/3607) and both methods combined (6.7%, 240/3607). Nearly three quarters of patients (72.5%, 2616/3607) had a previous history of self-harm.

A total of 2540 (70.4%) patients were admitted to a medical bed, 2333 (64.7%) received a psychosocial assessment, 103 (2.9%) were admitted to a psychiatric inpatient bed and 783 (21.7%) were referred to specialist mental health services in the community following hospital discharge.

### Clinical care and deprivation

There was some evidence that levels of clinical care varied between patients depending on the IMD score of their area of residence. Those from the least deprived areas were more likely to receive each of the elements of care examined (Table 1). The evidence for differences between IMD quintiles in the proportion of people receiving the different elements of care was strongest for receipt of psychosocial assessment and psychiatric inpatient admission, while it was weakest for community mental health follow-up and medical admission.

**Table 1:** Proportion of self-harm patients receiving medical admission, psychosocial assessment, psychiatric inpatient admission and community mental health follow-up by quintile of deprivation

	Medical admission *n* (%)	Psychosocial assessment *n* (%)	Psychiatric inpatient admission *n* (%)	Community mental health follow-up *n* (%)
Indices of multiple deprivation quintile				
1 (most deprived)	499 (70.58)	449 (63.51)	17 (2.40)	153 (21.64)
2	500 (70.52)	460 (64.88)	16 (2.26)	151 (21.30)
3	516 (69.54)	457 (61.59)	13 (1.75)	165 (22.24)
4	491 (68.29)	455 (63.28)	38 (5.29)	131 (18.22)
5 (least deprived)	534 (73.15)	512 (70.14)	19 (2.60)	183 (25.07)

The odds of receiving these elements of care by levels of deprivation were further investigated in multivariable logistic regression models (Table 2).

**Table 2:** Crude and adjusted odds of medical admission, psychosocial assessment, psychiatric inpatient admission and community mental health follow-up in self-harm patients by level of deprivation

Outcome	Deprivation quintile	Crude			Adjusted^a^		
		OR	95% CI	*p*	OR	95% CI	*p*
Medical admission	1 (most deprived)	1.00	–	–	1.00	–	–
	2	1.00	0.81–1.23	0.979	1.01	0.81–1.26	0.907
	3	0.95	0.76–1.19	0.660	1.03	0.82–1.30	0.777
	4	0.90	0.71–1.14	0.375	1.01	0.80–1.29	0.906
	5 (least deprived)	1.14	0.91–1.41	0.257	1.25	0.98–1.59	0.068
*p* for linear trend		0.572			0.107		
Psychosocial assessment	1 (most deprived)	1.00	–	–	1.00	–	–
	2	1.06	0.86–1.32	0.585	1.09	0.87–1.36	0.471
	3	0.92	0.73–1.17	0.497	0.96	0.75–1.23	0.774
	4	0.99	0.73–1.34	0.950	1.06	0.78–1.44	0.719
	5 (least deprived)	1.35	1.08–1.68	0.008	1.45	1.15–1.82	0.002
*p* for linear trend		0.054			0.013		
Psychiatric inpatient	1 (most deprived)	1.00	–	–	1.00	–	–
	2	0.94	0.43–2.05	0.871	0.90	0.40–2.00	0.795
	3	0.72	0.33–1.57	0.415	0.67	0.30–1.49	0.328
	4	2.26	1.01–5.09	0.048	2.08	0.92–4.75	0.080
	5 (least deprived)	1.08	0.53–2.23	0.825	0.97	0.45–2.06	0.928
*p* for linear trend		0.139			0.237		
Community mental health follow–up	1 (most deprived)	1.00	–	–	1.00	–	–
	2	0.98	0.76–1.27	0.877	0.99	0.76–1.29	0.939
	3	1.04	0.80–1.33	0.787	1.05	0.81–1.35	0.718
	4	0.81	0.59–1.10	0.173	0.80	0.58–1.09	0.163
	5 (least deprived)	1.21	0.94–1.56	0.137	1.27	0.98–1.65	0.069
*p* for linear trend		0.465			0.348		

^a^Adjusted for age, sex, method of self-harm and previous self-harm

The adjusted odds of medical admission (OR 1.25, CI 0.98–1.59, *p* = 0.068), psychosocial assessment (OR 1.45, CI 1.15–1.82, *p* = 0.002) and referral to community mental health follow-up (OR 1.27, CI 0.98–1.65, *p* = 0.069) were all greatest in patients from areas in the least deprived quintile. However, statistical evidence of a linear dose response between quintile of deprivation and odds of receipt of treatment only remained for psychosocial assessment once other explanatory factors (age, gender, method of self-harm and previous history of self-harm; see Table 2) had been controlled for.

For a small sub-group of the study population who received a psychosocial assessment (*n* = 911), Beck suicide intent scores were available [10]. The mean score was 8.7 in people from the most deprived areas and 9.5 in people from the least deprived. Ordinary least squares linear regression provided no evidence (likelihood ratio test *p* = 0.770) of an association between suicidal intent score and area-level deprivation, indicating the suicidal intent of people did not vary between quintiles of deprivation.

### Sensitivity analysis

#### Mediating effects of self-discharge

In our fully adjusted analysis, psychosocial assessment appeared to be the only element of care which was associated with deprivation. We investigated this further by exploring the effects of self-discharge on this association. People who present to hospital for self-harm are initially triaged in the emergency department by medical staff and subsequently referred to liaison psychiatry for a specialist mental health assessment. People who self-discharge prior to, or shortly after being triaged are less likely to receive an assessment as they spend less time in hospital. This is demonstrated by the fact that rates of psychosocial assessment were 12.5% in patients who self-discharged compared to 71.2% in patients who did not self-discharge. Self-discharge was also more common (14.4%) in people from the most deprived quintile compared to the least deprived quintile (7.81%; *χ* ^2^ = 17.6, *df* = 4, *p* = 0.001). As self-discharge is on the causal pathway between presentation to hospital and receiving mental health assessment, we treated this variable as a possible mediating factor.

Subsequent mediation analysis suggested 65.9% of the total effect of deprivation on psychosocial assessment was mediated through self-discharge. The indirect effect estimate suggested a unit increase in IMD quintile resulted in a 5% increase in the odds of psychosocial assessment (OR 1.05, CI 1.03–1.08, *p* < 0.001) while evidence for the direct effect (independent of self-discharge) was weaker (OR 1.03, CI 0.98–1.07).

#### Missing data and out-of-area presentations

We used multiple imputation to re-run the models and assess the validity of our findings taking account of patients with missing data. The findings from our imputed datasets produced similar effect estimates to our main analysis focusing on patients with complete data (see Supplementary Table S1). The imputed dataset suggested patients from the least deprived quintile had a 46% greater odds (OR 1.46, CI 1.19–1.79, *p* < 0.001) of receiving an assessment compared to patients presenting from the most deprived quintile.

Furthermore, including the 86 patients who had presented from communities not served by the hospital in our analysis did not alter our findings. There was still strong evidence of an association between quintile of deprivation and receipt of psychosocial assessment (OR for assessment in least deprived quintile: OR 1.46, CI 1.15–1.82, *p* = 0.001).

## Discussion

### Main findings

Socioeconomic deprivation is associated with an increase incidence of self-harm but its impact on the delivery of clinical care for self-harm patients is less well understood. We found variation across quintiles of socioeconomic deprivation in the proportion of people receiving the different elements of clinical care investigated. People from the least-deprived areas tended to be more likely to receive medical admission, psychosocial assessment, psychiatric inpatient admission and referral to community mental health services. Following adjustment for key confounding variables, including method of self-harm, psychosocial assessment in particular appeared more likely in people from less deprived areas. Two-thirds of the disparity in psychosocial assessment between deprivation quintiles was explained by the mediating effects of self-discharge, with people from more deprived areas being more likely to self-discharge than people presenting from less deprived areas.

Our findings suggest that the likelihood of psychosocial assessment, but not the other elements of self-harm clinical care, vary depending on the level of deprivation of the area the patient is presenting from. This finding is at odds with the fact that psychosocial assessment is the only element of clinical care investigated which is recommended for all self-harm patients by NICE guidelines [8]. Prompt triage and referral for a full psychosocial assessment of a patient’s needs and risk is a cornerstone of self-harm patient management. A growing body of evidence suggests those self-harm patients who receive a psychosocial assessment have a lower risk of having a repeat hospital presentation [3, 4]. This socioeconomic inequality in clinical care is, therefore, of particular concern, especially considering repeat self-harm is robustly associated with increased risk of suicide [11].

The incidence of self-harm and suicide both have well described positive associations with area levels of socioeconomic deprivation [12, 13]. However, there is some evidence to suggest that risk of death within the self-harm patient population is also socially patterned. Long-term follow-up of over 30,000 self-harm patients from the multicentre study for self-harm in England suggested all-cause mortality was greater in self-harm patients during the 6 years following hospital presentation for self-harm (standardised mortality ratio: 3.6, CI 3.5–3.8) when compared to the general population, however, this elevated risk was almost twofold higher in those patients from the most socioeconomically deprived areas [14]. Socially patterned levels of clinical care should be addressed if the disproportionate burden of disease in self-harm patients who are from more deprived areas is to be reduced.

The identified socioeconomic inequalities in the psychosocial assessment of self-harm patients seem to be related in large part to self-discharge. Those patients from less-deprived areas appeared more likely to remain in hospital, thereby maximising the timeframe for liaison psychiatry services to assess their mental health needs. People who self-harm and discharge themselves from hospital without a psychosocial assessment have been highlighted as a high risk group with often a high prevalence of previous self-harm [15], the strongest risk factor for repeat self-harm [16]. In-depth qualitative research could help to identify the factors driving the association between socioeconomic status and the receipt of clinical care for self-harm In turn, this may help to ensure that good quality care for self-harm patients is equally provided to all and that its provision is not determined by a patient’s socio-economic background.

In addition to understanding why people from more deprived areas are less likely to stay in hospital, it is important to also consider what other factors are increasing the likelihood of optimal care in people from less deprived areas. Sub-group analysis of Beck suicide intent scale scores suggested that the severity of self-harm cases did not vary according to area-level deprivation. Therefore, the improved clinical care received by patients from the least deprived areas does not appear to be related to greater clinical severity. Alternative explanations could be related to varying levels of resources between patients. Such resources may include education, verbal skills and the ability to understand complex information. These resources can be collectively termed cultural health capital [17]. The greater availability of these resources in people from less deprived areas may lead to clinical encounters more likely to result in optimal clinical care [17].

### Strengths and limitations

To our knowledge, this is the first investigation of the association of socioeconomic deprivation and the clinical care of self-harm. Key strengths are the use of robust, prospectively collected data from a bespoke registry system. Routine UK hospital admission statistics underestimate the incidence of hospital presenting self-harm by up to 60% as many patients are discharged directly from A&E [18] and so registry data are key in providing a comprehensive picture of the clinical epidemiology of self-harm. Not only do these data give a more accurate estimate of disease burden but they also allow a detailed description of the clinical care a self-harm patient receives. We were, therefore, able to investigate a number of key elements of self-harm patient care including receipt of a psychosocial assessment.

A number of important limitations should be considered when interpreting these data. Our findings are based on data derived from a single inner-city hospital and may not be generalizable to other populations. Indeed, previous retrospective cohort studies have suggested that an urban location of a hospital is associated with an increased likelihood of self-discharge [19]. It is, therefore, possible that less urbanised hospitals with lower levels of self-discharge may not experience socially patterned variation in clinical care for self-harm. Furthermore, self-harm patient clinical care has been shown to vary considerable between hospitals. The proportion of patients admitted in our study was 70%, while in a random sample of hospitals in England, this proportion has been shown to vary from 22 to 85%. Given this variation in practice between hospitals, caution should be used when generalising the results from this analysis to other centres [20]. Our analysis is further limited by the ecological nature of our exposure. We measured levels of deprivation at a LSOA level and our findings may suffer from ecological bias, i.e. they may not apply at the individual level.

## Summary

Our findings suggest levels of clinical care appear to vary depending on the level of deprivation in the area a patient is presenting from; patients from the least deprived areas are the most likely to receive NICE recommended psychosocial assessment. This socially patterned variation in care appears to be driven in large part by increased rates of self-discharge in patents from more deprived areas. These results require replication in other samples. Moreover, assuming our findings are replicable, we need to better understand why those from less-deprived backgrounds are more able to access optimal care.

## Electronic supplementary material

Below is the link to the electronic supplementary material.

Supplementary material 1 (DOCX 11 kb)
