# Supplementary material for: Socioeconomic deprivation and the clinical management of self-harm: a small area analysis
Source: Soc Psychiatry Psychiatr Epidemiol. 2017 Oct 4;52(12):1475–81. doi: 10.1007/s00127-017-1438-1 (PMC5702367; doi:10.1007/s00127-017-1438-1)
Supplement: Supplementary file 1 — Supplementary material 1 (DOCX 11 kb) [file 127_2017_1438_MOESM1_ESM.docx]

| Table S1. Adjusted odds of psychosocial assessment in self-harm patients by level of deprivation. | | | | |
| --- | --- | --- | --- | --- |
| Outcome | Deprivation quintile |  |  |  |
|  |  | Adjusted^a^ | | |
|  |  | OR | 95% CI | P |
| Psychosocial assessment | 1 (most deprived) | 1.00 | - | - |
|  | 2 | 1.09 | 0.89-1.33 | 0.414 |
|  | 3 | 0.99 | 0.81-1.21 | 0.937 |
|  | 4 | 1.19 | 0.97-1.46 | 0.093 |
|  | 5 (least deprived) | 1.46 | 1.19-1.79 | 0.000 |
| *p for linear trend* | | p<0.001 | | |
| a) Adjusted for age, sex, method of self-harm and previous self-harm. Data from multiple imputation analysis | | | | |
